# The Story of the Insane from Year to Year

**Published:** 1891-12-26

**Authors:** 


### The Story of the Insane from Year to Year.

### THE NOTTINGHAM COUNTY ASYLUM.

Unlike most of the asylums whose reports we have noticed, this one arranges its statistics from the 1st of April to the 31st of March. On the former date the asylum contained 300 patients, and on the latter the number had risen to 330. During the twelve months 117 patients were admitted, 32 recovered, and 38 died. Thrown into percentages, these figures yield 27·35 and 11·98 respectively. Table X., which shows the causes of insanity in those admitted, tells us that drink and hereditary influence account for 64 out of the 117 admissions. Verily, this is a terrible satire on our boasted civilization. Of the deaths, thirteen were caused by brain diseases, and eight by phthisis, while other lung diseases played some part in eight. Dr. Aplin once more emphasizes the importance of early treatment: "A mistaken kindness often prompts the relatives of an insane person to keep him or her at home as long as they can possibly do so, and then, when all hope is gone, the patient is sent most likely in a confirmed state of mental ill-health, not to be cured, but to be taken care of." While speaking of the inheritance of insanity, Dr. Aplin has some very sensible remarks which we regret being unable to quote *in extenso*, but we extract the following, and hope it may sink deep into the minds of our readers: "If it were better known, I think many marriages which result disastrously would be avoided. In connection with this subject various difficult social problems suggest themselves which need not be here discussed, but I must enter my protest against the deliberate propagation of insanity which one is constantly hearing of in lunacy practice." No doubt the problems are difficult, but if those who really know the danger of these marriages refrain from speaking out, and that with no uncertain voice, it may be long before we see much improvement. Scientific appliances are invented for the better investigation of insanity; thepharmacopœia is ransacked in search of something that will cure it; but we hear next to nothing of prevention, and yet this very report under review shows us that fifty per cent. of the patients sent to the Nottingham County Asylum had no business to exist at all. We are like children chasing a shadow, while the substance is at our feet. The Visiting Committee say: "The state and condition of the asylum is satisfactory, and its management, and conduct of the officers and servants and their care of the patients, are such as to meet with our approval." The Commissioners in Lunacy who recommended the removal of the asylum, say: "The necessity for considerable outlay here in such improvements as the structure and site permit is great. At present the asylum may be sufficient for custody, but the proper treatment of the patients, according to the views now generally entertained upon this subject, is, if not impracticable, a task of the utmost difficulty." There is a balance of £209 in favour of the farm, but no rent is charged, nor is the method of valuation given. The weekly cost per head is 9s. 7⅛d.

### THE CHESHIRE COUNTY ASYLUM AT MACCLESFIELD.

At the end of the year there were 18 patients additional to the numbers on January 1st, the numbers having risen from 565 to 583. The admissions reached 122, the recoveries 42, and the deaths 43. The percentage of recoveries was 36·9, and the deaths 7·5. Hereditary taint and drink account respectively for 27 and nine of the admissions. Twenty-one of the deaths were caused by brain diseases, and five by phthisis. When referring to the increase of the numbers of his patients, Dr. Sheldon has the following remarks, which, it is to be hoped, his committee will lay to heart: "The accumulation of the insane is one of the gravest matters with which county authorities have to deal, and I feel bound to bring it prominently before the attention of your Committee. The Upton Asylum appears to be full, so that the brunt of the increase in coming years will fall upon this asylum, the probability is that in eight years our accommodation will be exhausted; if so, in five or six years' time the matter will be pressing, and I cannot too strongly urge the importance of so using this breathing space, that when the crisis arrives some well-considered scheme may be ready." During the past five years post-mortem examinations were made in 246 cases out of 258 deaths, and Dr. Sheldon assumes some credit for this. In our opinion more credit would have been done to the medical staff had the post-mortem examinations been fewer. We doubt whether these examinations should ever be made (unless some great doubt exist as to the cause of death) without the willing consent of the deceased's friends, and we further doubt whether the friends of 246 patients, out of a total of 258, are likely to give this willing consent. Of course, the inhabitants of Cheshire may be exceptional in their zeal for pathology, and we have no right to make too sweeping assertions on the subject, and we sincerely hope Dr. Sheldon may not, some day, get into trouble over these post-mortems. The following paragraph on the "changes in the staff" is worthy of transcription: "To anyone acquainted with the conditions, such restlessness is not surprising, the danger attending the occupation, the often repulsive duties, the long hours, the small pay—which, in the case of men, almost prohibits marriage—the heavy responsibilities, and the uncertainty of superannuation, all operate against long service and efficiency." Some of these drawbacks are, from the nature of things, irremediable; but the long hours may be shortened a little; the pay may be increased; and, above all, the pensions made secure. When these have been done the services of suitable people will be easily enough found. If the report should fall into the hands of any farmer accustomed to grumble a little about the hard times, he will probably change his tune when he learns that a profit of £702 was last year made; and he will be anxious to try the no-rent system, with a sprinkling of free labour thrown in. The weekly cost of maintenance was 8s. 0⅞d.

